# Effects of a 6-month rowing training program in breast cancer survivors

**DOI:** 10.1371/journal.pone.0317118

**Published:** 2025-01-22

**Authors:** Mateo Real-Pérez, José Carlos Fernández-García, Juan Gavala-González

**Affiliations:** 1 Researching in Sport Science: Research Group (CTS-563) of the Andalusian Research Plan, University of Malaga, Malaga, Spain; 2 Department of Didactics of Languages, Arts and Sport, Andalucía-Tech, IBIMA, University of Málaga, Málaga, Spain; 3 Department of Physical Education and Sports, University of Seville, Seville, Spain; Amazonas State University, BRAZIL

## Abstract

The purpose of this longitudinal study was to determine the effects of a rowing training program on physical fitness and body composition in female breast cancer survivors (56.78 ± 6.38 years). Over six months, the participants (n = 40) performed two training sessions per week for an average of 75 minutes, combining resistance and muscle strength exercises with rowing. To evaluate the impact of the program, physical fitness tests and anthropometric measurements were carried out before and after the training protocol. The results show statistically significant improvements in measures such as weight (-1.84 kg), BMI (-0.7 kg/m2), hip (-3.24 cm) and waist circumference (-2.45 cm). In addition, variables associated with physical fitness also improved including leg strength (3.04 cm), dominant handgrip strength (4.24 kgf), non-dominant handgrip strength (3.49 kgf), aerobic endurance (78.35 m) and muscle flexibility (3.96 cm). We can therefore conclude that a rowing-based exercise program is an effective strategy to improve physical fitness in this type of population, yielding improvements in both anthropometric measurements and basic physical capacity variables, essential factors for better health and quality of life.

## Introduction

Cancer is a devastating disease that affects millions of people around the world, leaving in its wake not only medical, but also physical and psychological challenges [1]. Currently, breast cancer is the most prevalent cancer in women globally, with an estimated prevalence of 2.2 million new cases per year, accounting for 25% of cancer diagnoses in this population group [2, 3].

Although breast cancer survival rates have improved significantly in recent decades due to advances in detection, diagnosis, and treatment [4], the impact on women who have survived the disease often results in physical sequelae such as an increased perception of cancer-related fatigue [5] or the loss of muscle strength and aerobic capacity [6], as well as emotional challenges related to symptoms of depression, perceived self-image and self-esteem [7]. Furthermore, despite the great progress that has been made to date in the development of new, more specific and effective treatments for this disease, it has yet to be fully controlled [8].

In this context, physical activity has been recognized as an integral component in the process of recovery and improving quality of life for female breast cancer survivors [2, 9]. Evidence shows that maintaining adequate levels of physical activity can contribute to reducing cancer-associated symptoms of pain, fatigue and depression [10, 11]. In addition, a controlled training program can provide benefits in fitness-related variables such as endurance [12], muscle strength [13] and maintaining adequate body composition values [13–15]. Furthermore, studies suggest that reducing the time spent in sedentary activities and increasing physical activity can reduce cancer relapse and improve survival rates [2]. Taking this into account, the exercise guidelines promoted by the American College of Sports Medicine recommend maintaining or increasing rates of moderate physical activity to 150 minutes per week, including muscle strength training [16–18].

However, although the potential benefits of physical activity are widely recognized, the onset of complex diseases, such as breast cancer, may negatively influence physical activity levels in these patients, mainly due to uncertainty and lack of information [19]. Accordingly, in recent years rowing has been proposed as a type of non-invasive complementary therapy that can help to improve the quality of life in women breast cancer survivors [3]. The first studies carried out on rowing in women with breast cancer focused on analyzing the potential of this sport from the perspective of group therapy and its capacity to generate adherence to sports practice, a key factor in this type of population [20–22]. Subsequently, studies based on a 4-month intervention involving female breast cancer survivors have shown benefits in parameters related to health and quality of life, based on the results of questionnaires analyzing limitations in terms of mobility [3]. It has been shown that a 12-week rowing training program (three sessions per week, from 60 to 90 minutes in duration), significantly improves physical fitness variables (such as distance covered in the 6-minute walk test, or the results of flexibility and muscle strength tests), as well as body composition (total lean mass: +2.18 kg; and the percentage of total body fat: -2.63%) improving health and quality of life in women with breast cancer [13].

Our study examines the indications showing that the sport of rowing can be important in the planning and prescription of exercise programs in women with cancer and provides evidence confirming the importance of training as a complementary therapy during treatment. The choice of this sport over others is based on its cyclic and alternative actions of flexion and extension of the upper and lower limbs, while the stabilizing muscles of the trunk and back intervene during rowing to improve technique [23]. Although the existing literature on the influence of physical exercise and physical activity in breast cancer patients is increasingly relevant [24, 25], more studies are needed to determine which type of exercises should be recommended and how rowing can benefit people with this disease. For this reason, we believe our study represents an important advance in the prescription of healthy exercise for breast cancer survivors.

The aim of our study was to analyze the effects of a 6-month rowing training program on body composition and physical fitness in female breast cancer survivors.

## Materials and methods

### Participants

**Fig 1:**
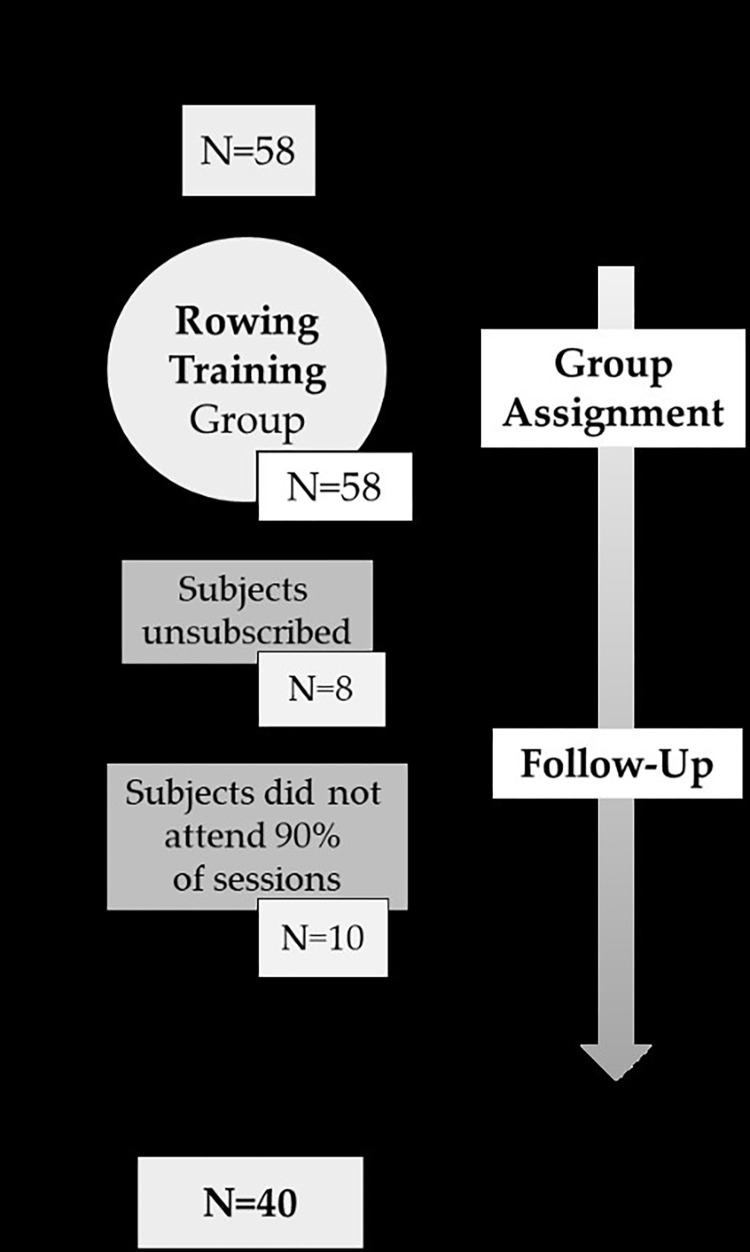
Flow diagram of the sample selected for this study.

All the participants (N = 40) who underwent the experiment had survived breast cancer (Table 1) and had the approval of their oncologist. After the initial selection, the nature of the study was explained to the participants, indicating that their anonymity would be maintained at all times, following the ethical considerations of Sport and Exercise Science Research [26] and the principles of the Declaration of Helsinki [27], which defines the ethical guidelines for research on human subjects. This study is registered with the Ethics Committee of the University of Malaga under No. 65-2020-H and date of approval:09/10/2020. The recruitment was from September, 5^th^ to December 22^nd^ 2022 (Fig 1). Informed consent was obtained from all subjects involved in the study; and written informed consent has been obtained from the patients to publish this paper. During the research, data were collected and processed and were treated following the Organic Law 3 3/2018, of 5 December, on Personal Data Protection and Guarantee of Digital Rights, regarding the protection of personal data under Spanish legislation.

**Table 1:** Descriptive analysis of the subjects.

	Age (years)	56.78 ± 6.38
	Years of diagnosis	6.58 ±5.72
*Body Composition*	Height (cm)	162.05 ± 5.59
	Weight (kg)	69.49 ± 9.48
	BMI (kg/m2)	26.48 ± 3.41
*Breast (%)*	Right	37.5
	Left	57.5
	Both	5.0
*Stage (%)*	I	7.5
	II	37.5
	III	40.0
	IV	15.0
*Surgery (%)*	Preservation	50.0
	Total Mastectomy	42.5
	Double Mastectomy	7.5
*Treatment (%)*	Quimiotherapy	93.3
	Radioterapy	93.3
	Tamoxifen*	100

*all patients currently have this treatment

### Instruments

Anthropometric data were collected using a Tanita BC 545N scale, with an accuracy of ±0.1 kg, for body composition and a SECA model 213 portable stadiometer, with an accuracy of ±1 mm, for height, following the manufacturer’s protocol for both clothing and food/liquid intake and the Frankfurt plane for head positioning.

A Cescorf anthropometric tape measure with an accuracy of ±1 mm was used to assess body circumference.

The tests and procedures described by Gavala et al. (2020) were used for the evaluation of physical fitness [28]: the sit-and-reach test for general flexibility (using the Baseline sit-and-reach box, with an accuracy of ±1 mm), the handgrip strength test for maximum isometric strength of the hand and forearm muscles (using a Takei 5401 dynamometer, with an accuracy of ±2 kgf) and the counter movement jump (CMJ) to measure lower body explosive strength (through the My Jump-2 application, with an accuracy of 1 cm). The 6-minute walk test was administered to evaluate endurance.

### Procedure

After the initial evaluation, the subjects underwent a consecutive 24-week rowing-based training program (Table 2). This program consisted of two 75-minute training sessions each week. These sessions were supervised by a trainer who monitored attendance (subjects who attended less than 90% of the sessions were excluded), the correct execution of the tasks and the intensity of the sessions. The training period was divided into three 8-week stages. These stages progressively increased in intensity and technical difficulty and were regulated through the participants’ subjective perception of effort, by means of the Börg scale [29].

**Table 2:** Design of the exercise prescription for the program.

Stage	Content	Duration
*1 Börg scale 5–6*.	Initial phase with mobility exercises, proprioceptive exercises and postural control exercises.	8’
	Main phase with rowing training.	60’
	Final phase with stretching.	7’
*2 Börg scale 6–7*.	Initial phase with mobility exercises, proprioceptive exercises and postural control exercises.	8’
	Main phase with rowing training.	60’
	Final phase with stretching.	7’
*3 Börg scale 7–8*.	Initial phase with mobility exercises, proprioceptive exercises and postural control exercises.	8’
	Main phase with rowing training.	60’
	Final phase with stretching.	7’

At the end of the 24-week training program, the participants were reassessed with the same procedure as in the initial evaluation.

### Statistical analysis

All analyses were performed with IBM SPSS Statistics, version 25. The level of significance was set at p < .05. The fit of the different variables to the normal distribution was assessed by both graphical procedures and the Shapiro Wilk test.

To analyze whether there were differences resulting from the rowing training, the differences between the means of each variable pre and post were analyzed and parametric Student’s t-tests were performed for related samples (paired data). Prior to this analysis, the normality of the distribution was checked using the Kolmogorov Smirnov test. In addition, a graphical analysis of the variables was performed using a box and whisker plot.

In addition, the effect size (η2p), which quantifies the size of the difference between groups, was calculated [30]. To calculate the effect size, the squared epsilon coefficient (ε2R) was determined. Effect sizes were reported using the Ɛ2 (small effect: Ɛ2 = 0.01, medium effect: Ɛ2 = 0.06; large effect: Ɛ2 = 0.14).

## Results

Table 3 shows the between-subject analysis of the study variables before and after the intervention program, as well as their evolution and comparisons to determine if there were statistically significant differences. The participants showed improvements in all the variables studied after 6 months of training.

**Table 3:** Analysis of the study variables before and after the intervention program.

	Pre-test (SD)	Post-test (SD)	ΔPre-Post (SD)	Student’s t	Effect size	p
**Body composition**						
**Weight (kg)**	69.49 (9.8)	67.65 (9.46)	-1.84 (1.6)	7.274	.253	.000**
**BMI (kg/m2)**	26.48 (3.58)	25.78 (3.46)	-.7 (.61)	7.264	.096	.000**
**Waist circumference (cm)**	87.82 (10.09)	84.57 (9.6)	-3.24 (2.44)	8.418	.385	.000**
**Hip circumference (cm)**	105.34 (8.07)	102.89 (7.95)	-2.45 (1.9)	8.138	.301	.000**
**Strength**						
**CMJ (cm)**	12.13 (3.28)	15.18 (3.29)	+3.04 (1.36)	-14.168	.215	.000**
**Dominant handgrip (kgf)**	21.59 (5.15)	25.83 (5.1)	+4.24 (2.32)	-11.57	.366	.000**
**Non-dominant handgrip (kgf)**	20.86 (5.04)	24.36 (5.34)	+3.49 (2.28)	-9.674	.361	.000**
**Aerobic capacity**						
**6-minute walk test (m)**	784.78 (103.64)	863.13 (108.75)	+78.35 (62.57)	-7.92	9.893	.000**
**Flexibility**						
**Sit-and-Reach (cm)**	1.23 (7.33)	5.19 (6.58)	+3.96 (3.18)	-7.875	.504	.000**

*p < .05; **p < .001

Regarding body composition, all the variables showed statistically significant improvements, both in weight (ΔWeightPre-Post:-1.84±1.6kg.; p = .000), as well as in BMI (ΔBMIPre-Post:-0.7±0.61), waist circumference (ΔWaist CircumferencePre-Post:-3.24±2.44cm; p = .000) and hip circumference (ΔHip CircumferencePre-Post:-2.45±1.9cm; p = .000). Analyzing the values associated with muscle strength, significant improvements were seen in the CMJ test (ΔCMJPre-Post = 3.04±1.36cm) and in upper limb strength, both in the dominant arm (ΔDominant-HGPre-Post:+4.24±2.32kgf; p = .000), and in the non-dominant arm (ΔNon-dominant-HGPre-Post:+3.49±2.28kgf; p = .000). In terms of endurance, the study subjects showed significant improvements in distance covered in the 6-minute walk test (Δ6-minute walk testPre-Post:+78.35±62.57m; p = .000). Finally, the subjects showed significant improvements in overall flexibility measured through the Sit-and-Reach (ΔSit-and-ReachPre-Post:+3.96±3.18cm; p = .000).

The changes in the study variables grouped according to the type of test (body composition, muscle strength, aerobic endurance and flexibility) and their evolution comparing before and after the training protocol can be observed graphically below, taking into account the values shown in Table 3.

**Fig 2:**
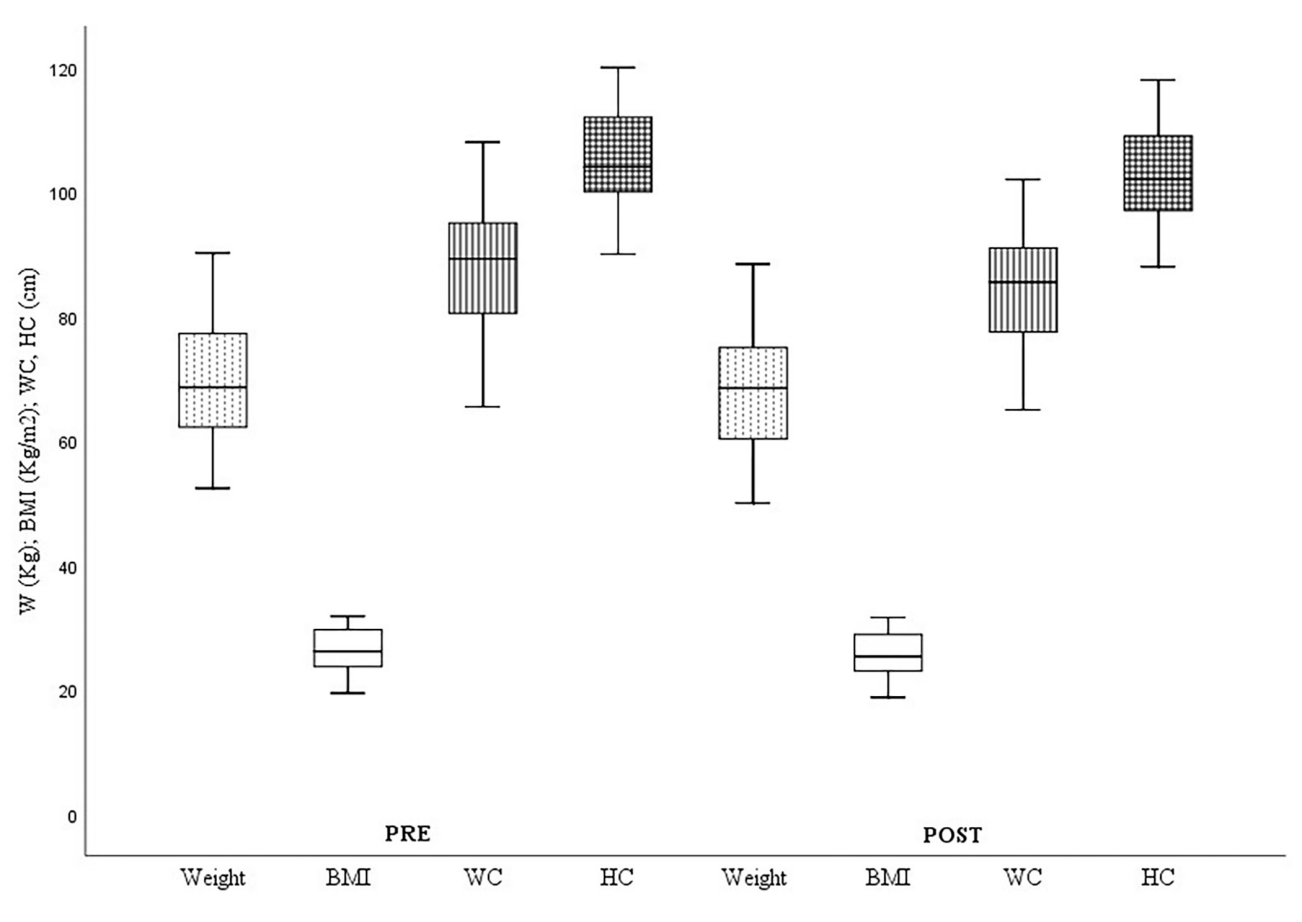
Anthropometry variables. Pre = pre-test; Post = post-test; W = weight; BMI = body mass index; WC = waist circumference; HC = hip circumference. *p < .05; **p < .001.

Anthropometric values showed significant improvements for all study variables (Fig 2), especially in waist (ΔWaist CircumferencePre-Post = -3.24±2.44cm.) and hip circumference (ΔHip CircumferencePre-Post = -2.45±1.9cm).

**Fig 3:**
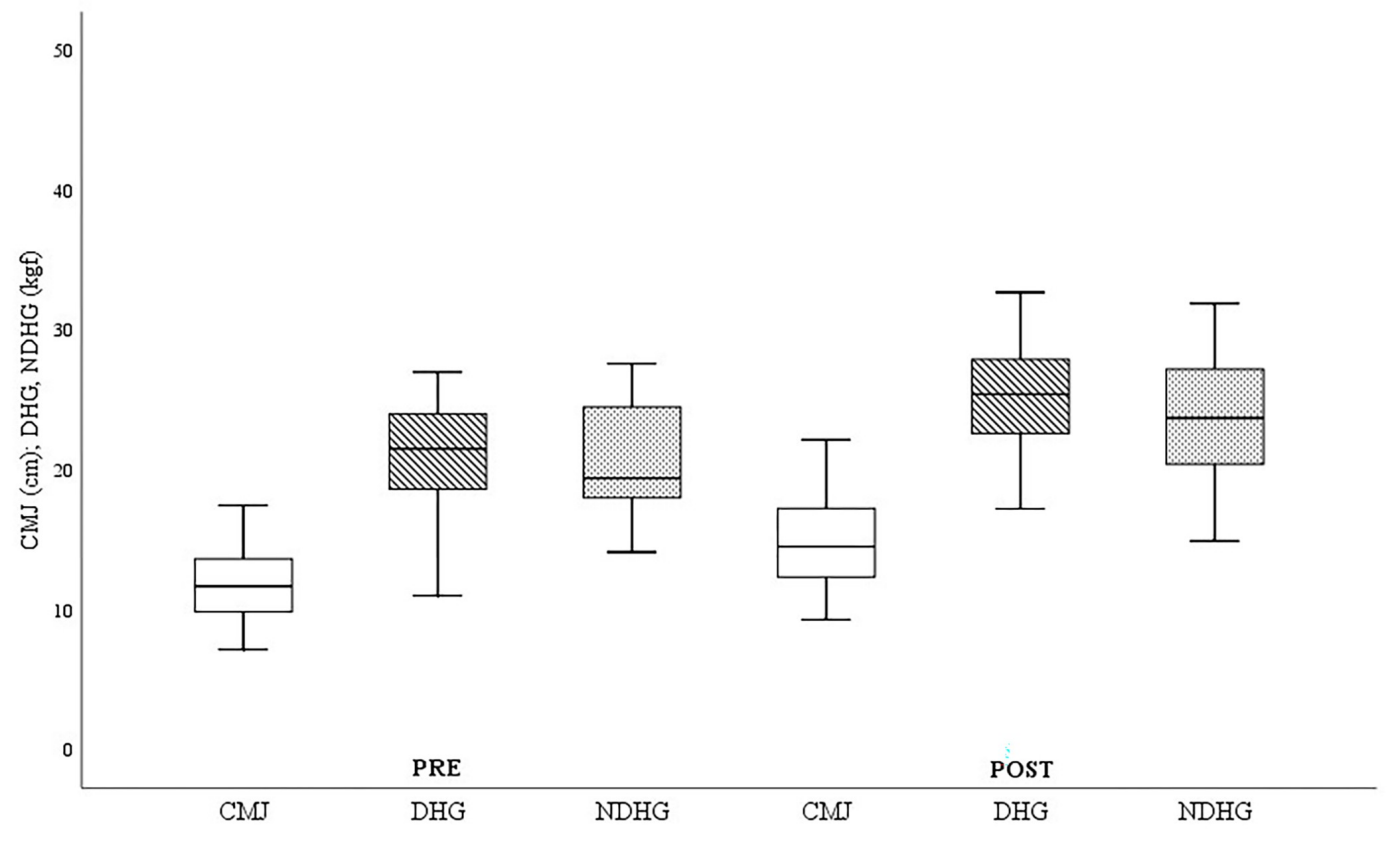
Strength variables. Pre = pre-test; Post = post-test; CMJ = counter movement jump; DHG = dominant handgrip; NDHG = non-dominant handgrip. *p < .05; **p < .001.

The analysis of the results associated with the strength variables (Fig 3) showed significant improvements after 6 months of training, both in the lower (ΔCMJPre-Post = 3.04±1.36cm) and upper limbs and in the dominant (ΔDominant-HGPre-Post = 4.24±2.32kgf) and non-dominant (ΔNon-dominant-HGPre-Post = 3.49±2.28kgf) arms.

**Fig 4:**
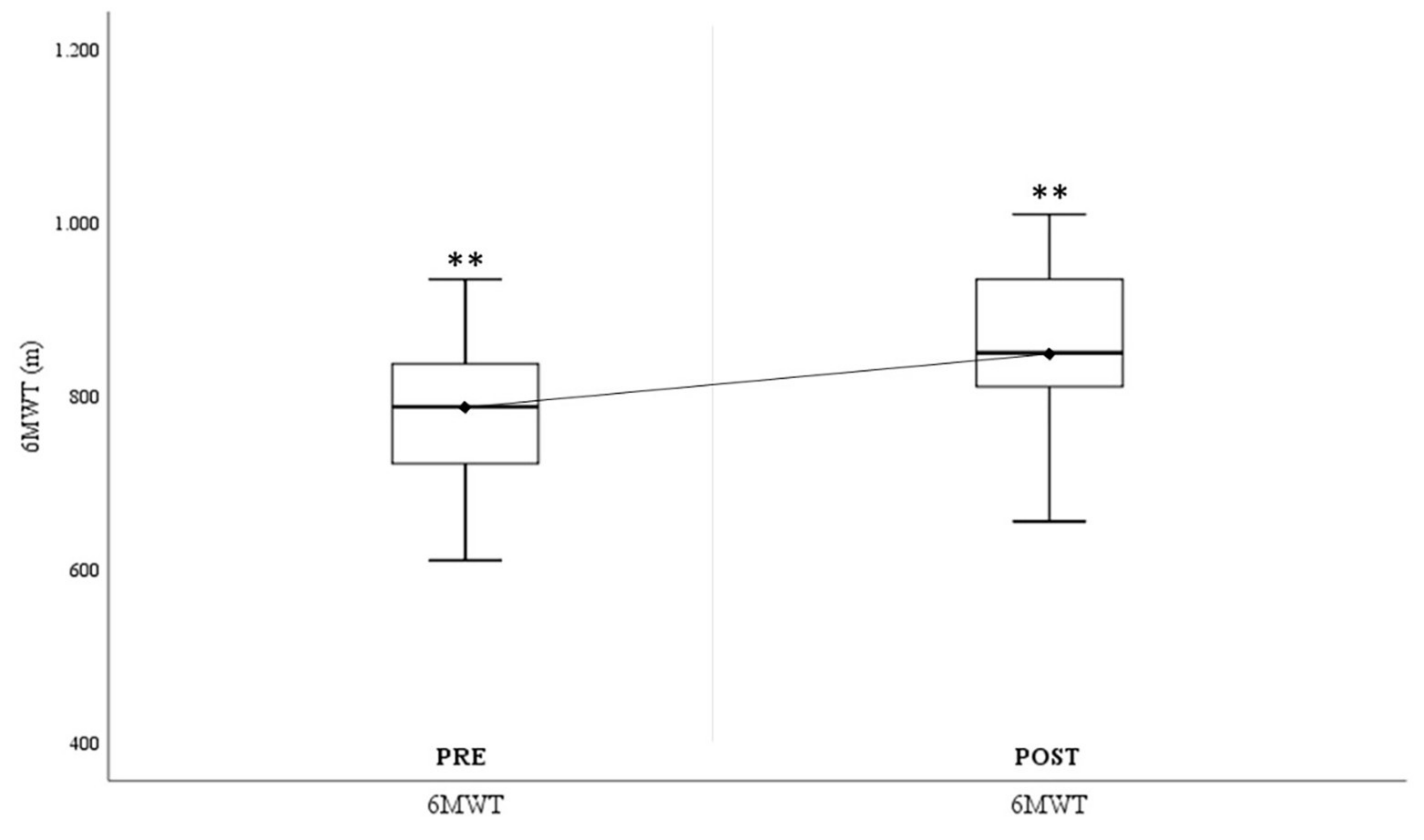
Aerobic capacity variables. Pre = pre-test; Post = post-test; 6MWT = 6-minute walk test. *p < .05; **p < .001.

Fig 4 illustrates the significant improvement in the distance covered in the 6-minute walk test (Δ6-minute walk testPre-Post = 78.35±62.57m.), showing an increase in the aerobic capacity of the study participants.

**Fig 5:**
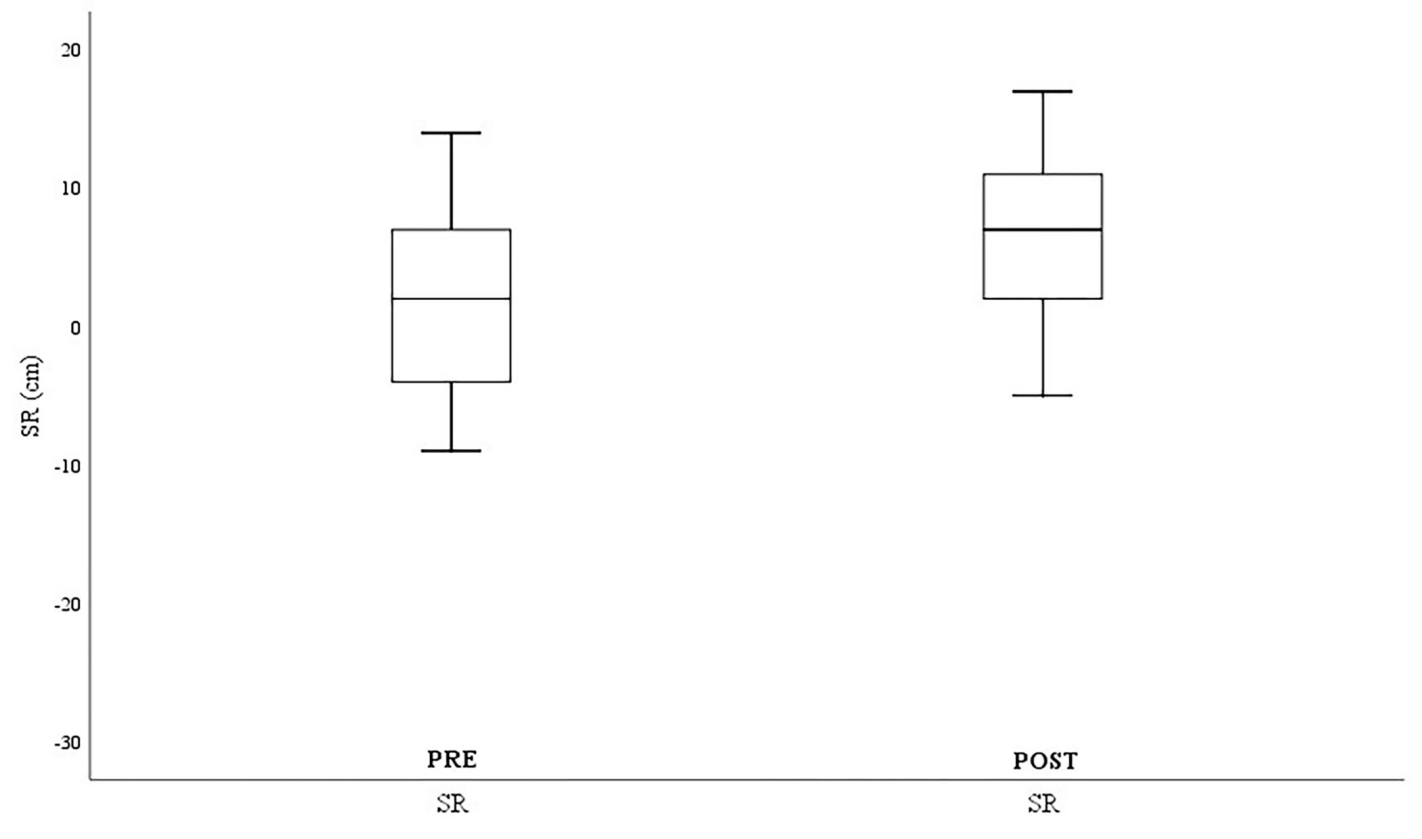
Flexibility variables. Pre = pre-test; Post = post-test; SR = Sit-and-Reach. *p < .05; **p < .001.

Finally, Fig 5 shows the changes in terms of overall flexibility, with significantly improved results in the Sit-and-Reach (ΔSit-and-ReachPre-Post = 3.96±3.18cm).

## Discussion

In recent years, the study of the potential influence of physical activity as a complementary therapy in women with breast cancer has been gaining relevance [31]. The process of managing cancer involves a structural reorganization of the person’s life. In this regard, studies [5, 11] have shown that maintaining adequate levels of physical activity can have a positive influence on recovery and survival rates, as well as prevent the decline caused by the disease itself and the currently established treatment techniques, improving the quality of life of people who are struggling with this situation. Therefore, the diagnosis of breast cancer should not be associated with discontinuing physical activity, but rather it should be a priority component that patients should integrate into their daily lives [9].

A review of the scientific literature reveals an increasing number of publications that support the importance of carrying out a training protocol that combines aerobic exercise with programs to improve muscle strength [4, 9, 12, 32, 33]. For example, multiple benefits have been reported after 16 weeks of training combining aerobic and strength exercises in women with breast cancer. These benefits are associated with improvements in cancer-associated symptoms such as fatigue and depression, as well as improvements in VO2 max, muscle strength and bone health, even in subjects who are physically inactive, overweight or obese and of different ethnic origins [12].

Furthermore, delving deeper into how physical activity can modulate breast cancer symptoms, we find reviews indicating that there are also positive changes in pro-inflammatory biomarkers associated with physical activity levels [5]. Studies in recent years have shown that physical activity is able to interact with different biological mechanisms typically altered in cancer such as inflammatory markers, sex hormones (estrogens and androgens), insulin and glucose levels (through the IGF-I insulin axis), adrenal hormones, vitamin D, the immune system, oxidative stress and DNA repair. As a result, the role of physical activity as a protector against cancer materializes in a decreased risk of carcinogenesis [19, 34–37].

In our study, we established a rowing-based training program in which we combined exercises involving aerobic capacity and muscle strength, due to the positive effect this sport can have on the physical recovery of female breast cancer survivors in terms of mobility and functionality [13]. After the 6 months of training, we observed statistically significant differences in the anthropometric parameters of the women participating in the study, such as a decrease in weight, BMI and waist and hip circumference, variables related to health and body composition.

In addition, in terms of the variables associated with physical fitness, we found significant improvements in various tests including aerobic capacity, muscle strength in the lower and upper limbs and general flexibility. This may help maintain physical function despite the decline caused by the disease itself and the current treatments, along with improving mobility and quality of life in female breast cancer survivors. These significant results not only coincide with those previously published in other longitudinal studies with shorter intervention times [28] but also surpass them, suggesting that training programs with a longer duration lead to greater improvements in variables associated with health and body composition, as well as those associated with physical fitness.

In short, based on the evidence found after carrying out the 6-month training protocol and comparing the results with other published studies [3, 13, 14, 28], we can affirm that rowing is a beneficial and safe activity for women breast cancer survivors. The characteristics of this sport foster the development of physical fitness and encourage participation through the influence of group therapy. Each boat is rowed by a team of women and a coxswain who steers the boat, and without their combined efforts and strength the boat would not be able to move forward [15].

Taking into account these scientifically proven benefits, we suggest that a rowing-based training protocol, guided by qualified professionals, contributes to maintaining adequate levels of physical activity in people with breast cancer. In addition, it can be a very important factor in promoting a healthy recovery and improving the quality of life of these women, thereby increasing their survival rate [6].

The strength of this pioneering study is the data it provides on physical activity and rowing in relation with physical condition, demonstrating that rowing is a viable and safe sport for breast cancer survivors. However, the study also has some limitations related to the sample power of the participants and the absence of a control group to compare de progress. Our findings are based on a relatively small sample, due to the very specific population encompassed by the study, the complexity in its recruitment and subsequent follow-up during the intervention protocol.

Despite the positive outcomes observed in this study, future research should focus on analyzing the biological effects of such training programs. This could help elucidate the physiological changes they may induce in relation to cancer in this specific population.

## Conclusion

The results have demonstrated the positive influence of a 6-month training program based on the sport of rowing, a cyclic and symmetrical activity that combines muscle strength with aerobic endurance, to improve parameters associated with anthropometric measurements, aerobic capacity, muscle strength and general flexibility in women breast cancer survivors. It was also found that a program of longer duration had more positive effects in all the variables studied.
